# PlGF, immune system and hypertension

**DOI:** 10.18632/oncotarget.4914

**Published:** 2015-07-18

**Authors:** Daniela Carnevale, Giuseppe Lembo

**Affiliations:** Department of Angiocardioneurology and Translational Medicine, IRCCS Neuromed, Pozzilli (IS) and Department of Molecular Medicine, “Sapienza” University of Rome, Rome, Italy

**Keywords:** placental growth factor, hypertension, immune System

The huge diffusion of hypertension and its associated complications has a significant impact on public health [1]. However, despite the high prevalence of essential hypertension and many efforts of research, the basic pathophysiological causes remain puzzling. Although in the simplest way, the determinants of blood pressure (BP) are approximated by physical laws for fluid dynamics, these components of BP and their interactions have been intensely examined for years, without untangling the core of the problem. A more recent field of studies in hypertension has begun to shed light on a previously unpredictable role of the immune system in regulating BP [2]. Nevertheless, even though it is now recognized that T cell response drives hypertension, the key immune pathways contributing to BP elevation are still unidentified.

Our recent work outlined an unprecedented role of immune system in the spleen, orchestrating the response of T cell to hypertensive challenge, through the angiogenic factor named Placental Growth Factor (PlGF) [3]. Two main points emerge in our study. First, the finding that PlGF-deficient mice manifest protection from AngII-induced hypertension, one of the most common experimental models of hypertension. Second, our work highlighted an unprecedented and unexpected role of the splenic neuroimmune interaction in hypertension. Although the fact that T cells are recruited in the vasculature during hypertension has been well documented, how and where they are activated was still unclear. In our paper, we have defined that the spleen is crucial in the onset of hypertension, given that splenectomized mice are protected from the blood pressure raising typically occurring upon chronic infusion of AngII. Moreover, the sympathetic drive converging in the spleen and departing from the celiac ganglion is necessary for the induction of PlGF in the MZ of splenic reservoir upon the AngII hypertensive challenge. Thus, we started to think about a “neuroimmune” pathway mediated by PlGF and necessary for the onset of hypertension. The generation of chimeric mice, obtained by spleen transplantation and harboring a PlGF-deficient spleen in a WT background and the contrary, allowed us to finally demonstrate that PlGF is needed in the spleen to allow the activation of T cells and blood pressure raising. Once activated in the spleen, PlGF is crucial to allow T cell costimulation via CD86 and their consequent egress for the deployment toward vasculature of target organs, a hallmark of hypertension. The identification of an epigenetic modulation, responsible for the PlGF-mediated mechanism of T cells costimulation, highlights intriguing questions that range over cardiology toward oncology. In particular, we found that the co-stimulatory molecule CD86 is regulated by Timp3 expression, depending in turn on the activity of Sirt1 deacetylase activity on p53 repressor activity.

Despite the unquestionable basic research implications that our results would have in the evergrowing field of studies on immune system and hypertension, what is more important is the opportunity arousing in terms of therapeutic potential. Indeed, the possibility to use potent immunosuppressive agents to treat uncomplicated hypertension would inevitably provoke some uncertainty. On the other hand, with the identification of key immune pathways contributing to BP elevation, specific therapeutic interventions might be devised to ameliorate hypertension, minimizing the potential immunosuppressive deleterious effects. On this issue, PlGF appears as an appealing molecular target for therapeutic implications, since tools clinically affording this pathway already exist [4, 5]. In the last years, anti-PlGF monoclonal antibodies have been developed as a strategy to slow tumor growth and for age-related macular degeneration. The ongoing clinical trials, testing humanized monoclonal antibodies directed to PlGF, encourage the possibility to endeavor this way also in hypertension and hopefully fulfill the pressing priority to reach satisfactory control rates in the substantial proportion of people with hypertension that, although under treatment, do not achieve the BP target levels recommended by current guideline.

**Figure:**
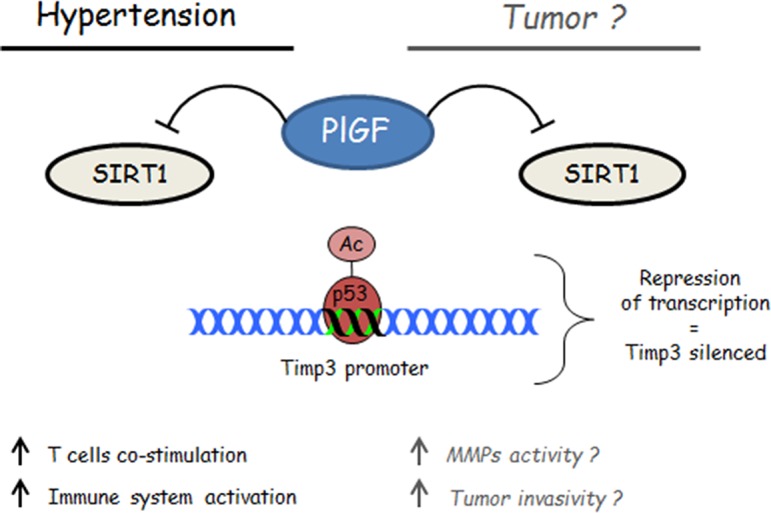


At same time, our findings encourage a reflection about the possibility that some of the effects so far attributed to PlGF inhibition in cancer may rely on the previously unknown capability to exert an epigenetic modulation of p53-Timp3 axis, which is well known to play a crucial role also in tumor growth. Further studies directed at this issue, could help to explain how a single molecule like PlGF could play so wide and, at same time similar, effects in different pathophysiological context, like hypertension and cancer.
